# Ninjin'yoeito Targets Distinct Ca^2+^ Channels to Activate Ghrelin-Responsive vs. Unresponsive NPY Neurons in the Arcuate Nucleus

**DOI:** 10.3389/fnut.2020.00104

**Published:** 2020-07-17

**Authors:** Chayon Goswami, Katsuya Dezaki, Lei Wang, Akio Inui, Yutaka Seino, Toshihiko Yada

**Affiliations:** ^1^Division of Integrative Physiology, Center for Integrative Physiology, Kansai Electric Power Medical Research Institute, Kobe, Japan; ^2^Division of Diabetes, Metabolism and Endocrinology, Kobe University Graduate School of Medicine, Kobe, Japan; ^3^Division of Integrative Physiology, Department of Physiology, Jichi Medical University School of Medicine, Tochigi, Japan; ^4^Department of Biochemistry and Molecular Biology, Bangladesh Agricultural University, Mymensingh, Bangladesh; ^5^Faculty of Pharmacy, Iryo Sosei University, Iwaki, Japan; ^6^Pharmacological Department of Herbal Medicine, Kagoshima University Graduate School of Medical & Dental Sciences, Kagoshima, Japan; ^7^Center for Diabetes Research, Division of Diabetes and Endocrinology, Kansai Electric Power Medical Research Institute, Kobe, Japan

**Keywords:** Ninjin-yoeito, anorexia, arcuate nucleus, neuropeptide Y, ghrelin, N-type Ca^2+^ channel, L-type Ca^2+^ channel

## Abstract

Appetite loss or anorexia substantially deteriorates quality of life in various diseases, and stand upstream of frailty. Neuropeptide Y (NPY) in the hypothalamic arcuate nucleus (ARC) and ghrelin released from stomach are potent inducers of appetite. We previously reported that Ninjin'yoeito, a Japanese kampo medicine comprising twelve herbs, restores food intake, and body weight in cisplatin-treated anorectic mice. Furthermore, Ninjin'yoeito increased cytosolic Ca^2+^ concentration ([Ca^2+^]_i_) in not only ghrelin-responsive but ghrelin-unresponsive NPY neurons in ARC. The cellular lineage/differentiation of ghrelin-unresponsive neuron is less defined but might alter along with aging and diet. This study examined the occupancy of ghrelin-unresponsive neurons among ARC NPY neurons in adult mice fed normal chow, and explored the mechanisms underlying Ninjin'yoeito-induced [Ca^2+^]_i_ increases in ghrelin-unresponsive vs. ghrelin-responsive NPY neurons. Single ARC neurons were subjected to [Ca^2+^]_i_ measurement and subsequent immunostaining for NPY. Ghrelin failed to increase [Ca^2+^]_i_ in 42% of ARC NPY neurons. Ninjin'yoeito (10 μg/ml)-induced increases in [Ca^2+^]_i_ were abolished in Ca^2+^ free condition in ghrelin-responsive and ghrelin-unresponsive ARC NPY neurons. Ninjin'yoeito-induced [Ca^2+^]_i_ increases were inhibited by N-type Ca^2+^ channel blocker ω-conotoxin in the majority (17 of 20), while by L-type Ca^2+^ channel blocker nitrendipine in the minority (2 of 23), of ghrelin-responsive neurons. In contrast, Ninjin'yoeito-induced [Ca^2+^]_i_ increases were inhibited by nitrendipine in the majority (14 of 17), while by ω-conotoxin in the minority (8 of 24), of ghrelin-unresponsive neurons. These results indicate that ghrelin-unresponsive neurons occur substantially among NPY neurons of ARC in adult mice fed normal chow. Ninjin'yoeito preferentially target N-type and L-type Ca^2+^ channels in the majority of ghrelin-responsive and ghrelin-unresponsive neurons, respectively, to increase [Ca^2+^]_i_. We suggest ARC N- and L-type Ca^2+^ channels as potential targets for activating, respectively, ghrelin-responsive, and unresponsive NPY neurons to treat anorexia.

## Introduction

Reduced appetite and body weight are associated with cancer, sarcopenia and frailty (1, 2), and deteriorate the quality of life (QOL) (3, 4). Hence, effective means to promote appetite have been awaited. Ninjin'yoeito, a Japanese traditional Kampo medicine, has been used clinically and demonstrated to be effective to treat anorexia, fatigue, anemia, cold limbs, persistent cough, mental disequilibrium, and to promote recovery from disease (5–7). Its ability to ameliorate this variety of symptoms is considered to result partly from counteraction of anorexia. Ninjin'yoeito comprises 12 crude drugs (Table 1). Some of these crude drugs are known to pass through the blood brain barrier (BBB) and hence possibly access to the hypothalamus including the arcuate nucleus (ARC), while ghrelin, an orexigenic gut hormone, freely accesses to the ARC neurons without crossing the BBB (8). Hence, oral administration of Ninjin'yoeito could act on ARC via one or both of these routes.

**Table 1:** Composition (daily dose^*^) of kampo formula Ninjin'yoeito.

**Ingredient**		**Contents (g)**
**English name**	**Latin name**	
Rehmannia root	*Rehmanniae radix*	4.0
Japanese angelica root	*Angelicae acutilobae*	4.0
Atractylodes rhizome	*Atractylodis rhizoma*	4.0
Poria sclerotium	*Poria*	4.0
Ginseng	*Ginseng radix*	3.0
Cinnamon bark	*Cinnamomi cortex*	2.5
Polygala root	*Polygalae radix*	2.0
Peony root	*Paeoniae radix*	2.0
Citrus unshiu peel	*Citri unshiu pericarpium*	2.0
Astragalus root	*Astragali radix*	1.5
Glycyrrhiza	*Glycyrrhizae radix*	1.0
Schisandra fruit	*Schisandrae fructus*	1.0

* *Approximate 6,700 mg of dried water extract of Ninjin'yoeito was prepared in GMP-standardized factory of Kracie Pharma, Ltd. (Japan) on the basis of above described composition*.

We previously reported that Ninjin'yoeito counteracted the anorexigenic and body weight-lowering effects of cisplatin, a chemo-therapy drug, in mice (9). In parallel, Ninjin'yoeito increased cytosolic Ca^2+^ concentration ([Ca^2+^]_i_) in single neurons isolated from ARC, and the majority (79%) of the Ninjin'yoeito-responsive neurons were immunoreactive (IR) to neuropeptide Y (NPY). The neuron coexpressing NPY and agouti-related protein (AgRP) (NPY/AgRP neuron) in ARC of hypothalamus is considered as the principal neuron for initiating feeding behavior (10–13). Selective activation of ARC NPY/AgRP neurons acutely and robustly triggers feeding (14, 15), while their selective deletion in adult mice markedly reduces feeding (16, 17), placing the ARC NPY/AgRP neuron as the necessary and adequate inducer of feeding.

We reported previously that Ninjin'yoeito increased [Ca^2+^]_i_ in the ghrelin-responsive and ghrelin-unresponsive NPY neurons in ARC. Ghrelin, released from gut under fasted condition, stimulates feeding via activating NPY neurons (18–20). Hence, the ghrelin-responsive NPY neurons in ARC is considered to play a central role in stimulating physiological feeding. Compared to ghrelin-responsive NPY neurons, the role of the ghrelin-unresponsive NPY neurons is less defined. A possibility exists that ghrelin-responsive and ghrelin-unresponsive NPY neurons could be converted to each other depending on the diet/metabolic states and aging, including ghrelin resistance (21–23). The present study firstly investigated the occupancy (percentage) of the ghrelin-unresponsive neurons among ARC NPY neurons in adult mice fed normal chow. Notably, it has been documented that ghrelin-resistance occurs with aging and likely contributes to appetite reduction and resultant BW decrease and frailty. In this line, our previous finding that Ninjin'yoeito interacts with and recruits ghrelin-unresponsive NPY neurons to [Ca^2+^]_i_ increases suggests its potential to restore appetite and counteract frailty. Hence, it is of relevance to elucidate the mechanisms underlying the [Ca^2+^]_i_ responses to Ninjin'yoeito in ghrelin-unresponsive NPY neurons. The present study aimed to elucidate the Ca^2+^ channel type implicated in the [Ca^2+^]_i_ response to Ninjin'yoeito in ghrelin-unresponsive NPY neurons in comparison with that in ghrelin-responsive NPY neurons.

It has been reported that N-type (CaV2.2) and L-type (CaV1.3) voltage-dependent Ca^2+^ channels (VDCCs) participate in depolarization-induced release of NPY in rat medium eminence-ARC preparation (24). In this report, 23 mM KCl-induced NPY release was blunted by nitrendipine, a blocker of L-type Ca^2+^ channels, and 45 mM KCl-induced NPY release was markedly attenuated by ω-conotoxin, a blocker of N-type Ca^2+^ channels, but not by nitrendipine. These data may indicate differential roles of N-type and L-type Ca^2+^ channels depending on the magnitude of depolarization by KCl. In addition, N-type and/or L-type Ca^2+^ channels in ARC neurons are involved in the [Ca^2+^]_i_ responses to several substances including ghrelin (25, 26). Hence, the present study explored the link of Ninjin'yoeito to N-type and/or L-type VDCCs in ghrelin-responsive vs. unresponsive neurons. Single neurons were isolated from ARC of adult mice fed normal chow, subjected to Ca^2+^ imaging to determine their responsiveness to ghrelin and Ninjin'yoeito, and subsequently immunostained for NPY. In the ghrelin-responsive and ghrelin-unresponsive NPY neurons, the effects of Ninjin'yoeito on [Ca^2+^]_i_ and their modulation by N-type and L-type Ca^2+^ channel blockers were examined.

## Materials and Methods

### Ninjin'yoeito Extract

Ninjin'yoeito is an herbal supplement composed of 12 crude drugs (Table 1). Ninjin'yoeito extract was supplied by Kracie Co. (Tokyo, Japan). Ninjin'yoeito extract was mixed with distilled water to prepare the stock solution.

### Materials and Solution

For [Ca^2+^]_i_ imaging, Ninjin'yoeito solution was diluted at the concentrations used for superfusion in HEPES-buffered Krebs-Ringer bicarbonate buffer (HKRB) solution composed of (in mM) 129 NaCl, 5.0 NaHCO_3_, 4.7 KCl, 1.2 KH_2_PO_4_, 1.8 CaCl_2_, 1.2 MgSO_4_, and 10 HEPES with pH adjusted at 7.4 using NaOH. Ghrelin was purchased from Peptide Institute (Osaka, Japan). Fresh solution of Ninjin'yoeito and ghrelin were prepared before each experiment.

### Animals

Male C57BL/6J mice aged 4–6 weeks were obtained from Japan SLC (Shizuoka, Japan) and housed for at least 1 week under conditions of controlled temperature (23 ± 1°C), humidity (55 ± 5%) and lighting (light phase 7:30–19:30). Food and water were available ad libitum. Animal experiments were carried out after receiving approval from the Institutional Animal Experiment Committee and in accordance with the Institutional Regulation for Animal Experiments at Jichi Medical University (IACUC approval number; 17-229) and Kobe University (IACUC approval number; 30-10-06-R1).

### Preparation of Single Neurons From ARC

The ARC was isolated from the brain of mice aged 5–7 weeks and single neurons were prepared as reported previously (25). Briefly, mice were anesthetized with intraperitoneal injection of urethane (ethyl carbamate; 1 g/kg, ip) or inhalation administration of isoflurane and decapitated, and their brain was removed. Brain slices containing ARC were prepared, and the whole ARC of the left and right sides was punched out. The dissected tissues were incubated in HKRB supplemented with 20 units/ml papain (Sigma Aldrich, St. Louis, MO), 0.015 mg/ml deoxyribonuclease, and 0.75 mg/ml BSA for 16 min at 36°C in a shaking water bath, followed by gentle mechanical trituration for 5–10 min. The cell suspension was centrifuged at 100 × g for 5 min. The pellet was resuspended in HKRB and distributed onto coverslips. The cells were kept at 30°C in moisture-saturated dishes till [Ca^2+^]_i_ measurements for up to 6 h.

### Measurements of [Ca^2+^] in Single ARC Neurons

At 2–10 h after cell preparation, [Ca^2+^]_i_ was measured by ratiometric fura-2 fluorescence imaging as previously reported (25). Briefly, following incubation with 2 μM fura-2AM (DOJINDO, Kumamoto, Japan) for 30 min at 30°C, the cells were mounted in a chamber and superfused at 1 ml/min with HKRB containing 2.5 mM glucose at 30°C. Data were taken from the single cells that were identified as neurons by the criteria reported previously (25); relatively large diameter (≥10 μm), clear and round cell bodies on phase-contrast microscopy. Ninjin'yoeito (10 μg/ml) and ghrelin (10^−8^ M) were administered under superfusion conditions. Fluorescence ratio (F340/F380) images were produced by Aquacosmos ver. 2.5 (Hamamatsu Photonics, Shizuoka, Japan). When [Ca^2+^]_i_ increases took place within 10 min of superfusion with agents and their amplitudes were at least twice larger than fluctuations of baseline, they were considered responses. In all experiments, neurons from at least three separate preparations were analyzed.

### Immunocytochemistry and Identification of NPY Neurons

After [Ca^2+^]_i_ measurements, cells were fixed with 4% paraformaldehyde, pretreated with 3% H_2_O_2_ for 1 h, and blocked in 10% normal goat serum and in 0.1 M PBS for 1 h at room temperature. Cells were incubated overnight at 4°C with primary antiserum to NPY (DiaSorin, Stillwater, MN) diluted 1:10,000 in PBS containing 1.5% normal goat serum. After rinsing, cells were incubated with biotinylated secondary antibody raised against rabbit IgG (Vector Laboratories Inc., Burlingame, CA; diluted 400-fold) for 1 h at room temperature. After rinsing, the sections were labeled with avidin-peroxidase complex (ABC kit, Vector) for 1 h and color-developed with 3, 3'-diaminobenzidine (DAB). [Ca^2+^]_i_ and immunocytochemical data were correlated to each other, based on the photographs of the single neurons subjected to [Ca^2+^]_i_ measurements in the microscopic field (25).

## Results

### Effect of Ghrelin on [Ca^2+^]_i_ in ARC NPY Neurons

**Figure 1:**
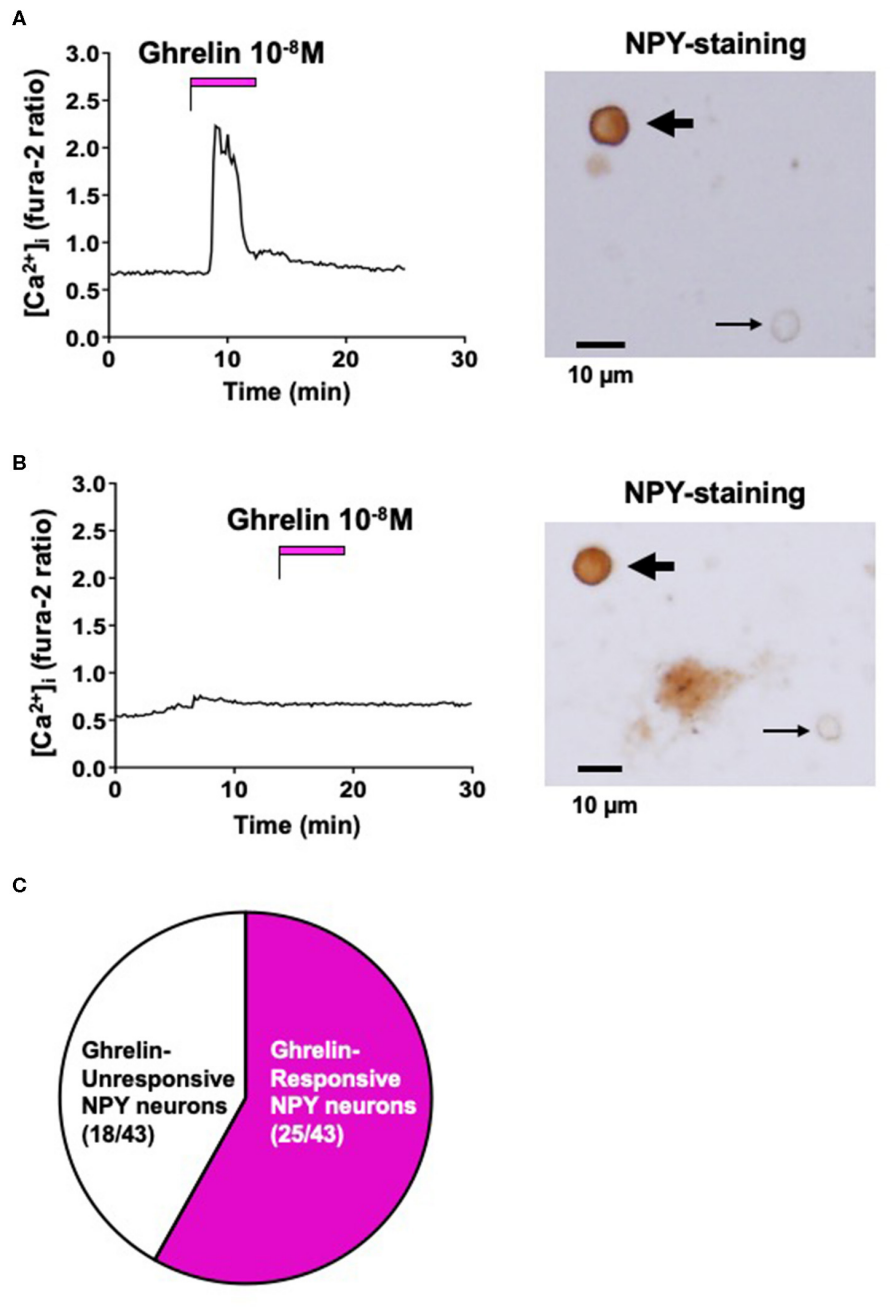
Effect of ghrelin on [Ca^2+^]_i_ in ARC NPY neurons. **(A)** Ghrelin (10^−8^ M) increased [Ca^2+^]_i_ in a single ARC neuron that was subsequently shown to be IR to NPY, indicated by the thick arrow in right. **(B)** Ghrelin (10^−8^ M) failed to increase [Ca^2+^]_i_ in a single ARC neuron that was subsequently shown to be IR to NPY, indicated by the thick arrow in right. Thin arrows in right of **(A,B)** indicate neurons that were not IR to NPY. Scale bars in right of **(A,B)** show 10 μm. **(C)** Incidence of ghrelin-responsive and ghrelin-unresponsive ARC NPY neurons: 25 of 43 (58%) neurons responded to ghrelin and 18 of 43 (42%) neurons did not respond to ghrelin. Glucose concentration was 2.5 mM. These data were obtained from 3 preparations of single neurons from 3 mice.

Single neurons isolated from ARC were superfused with HKRB containing 2.5 mM glucose for [Ca^2+^]_i_ imaging. After [Ca^2+^]_i_ was stabilized at baseline, administration of ghrelin (10^−8^ M) increased [Ca^2+^]_i_ in some single neurons that were subsequently shown to be immunoreactive (IR) to NPY by immunocytochemistry (Figure 1A). In contrast, administration of ghrelin failed to increase [Ca^2+^]_i_ in other single neurons that were IR to NPY (Figure 1B). Among 43 ARC NPY neurons, 25 (58%) neurons responded to ghrelin and 18 (42%) neurons did not respond to ghrelin (Figure 1C). These results indicated that ghrelin-unresponsive neurons occur substantially among NPY neurons of ARC in adult mice fed normal chow.

### Ninjin-yoeito Increases [Ca^2+^]_i_ via Ca^2+^ Influx Preferentially Through N-Type VDCC in the Minority of Ghrelin-Responsive Neurons

**Figure 2:**
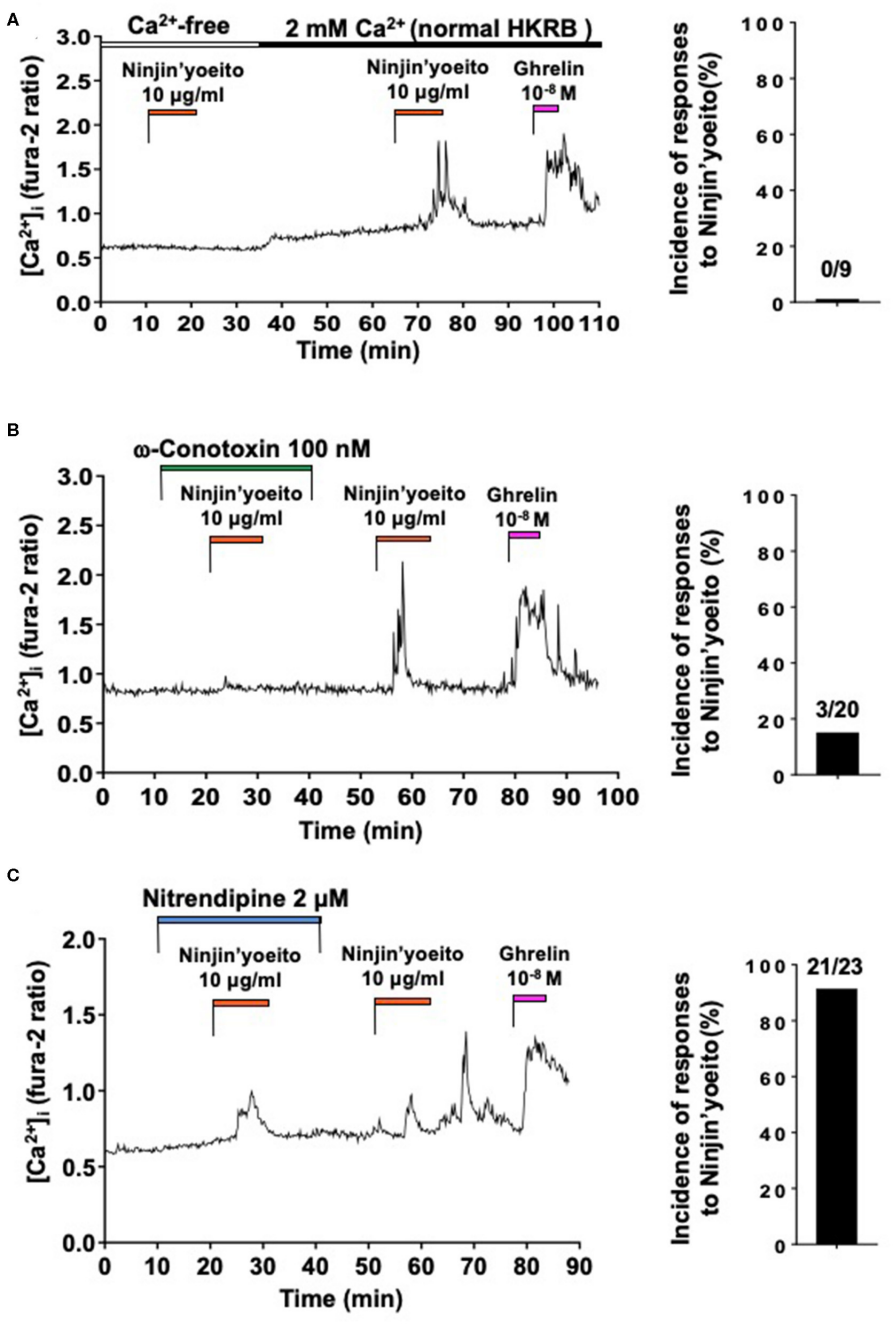
Ninjin'yoeito-induced [Ca^2+^]_i_ increases were inhibited in Ca^2+^ free condition and preferentially by N-type Ca^2+^ channel blocker in the majority of ghrelin-responsive ARC NPY neurons. ARC NPY neurons that responded to Ninjin'yoeito (10 μg/ml) and ghrelin (10^−8^ M) with increases in [Ca^2+^]_i_ were studied. Glucose concentration was 2.5 mM. **(A)** The [Ca^2+^]_i_ response to Ninjin'yoeito was inhibited under superfusion with Ca^2+^ free HKRB. Right: incidence (%) of responses to Ninjin'yoeito in Ca^2+^ free HKRB. Numbers above bar indicate number of neurons responding to Ninjin'yoeito over that examined. Data were from five preparations of single neurons from four mice. **(B)** An N-type Ca^2+^ channel blocker, ω-conotoxin, inhibited the [Ca^2+^]_i_ response to Ninjin'yoeito. Right: incidence of responses to Ninjin'yoeito in the presence of ω-conotoxin. Numbers above bar indicate number of neurons responding over examined. Data were from six preparations of single neurons from three mice. **(C)** An L-type Ca^2+^ channel blocker, nitrendipine, failed to inhibit the [Ca^2+^]_i_ response to Ninjin'yoeito. Right: incidence of responses to Ninjin'yoeito in the presence of nitrendipine. Numbers above bar indicate number of neurons responding over examined. Data were from 9 preparations of single neurons from four mice.

The effect of Ninjin'yoeito (10 μg/ml) on [Ca^2+^]_i_ in ARC NPY neurons that responded to ghrelin were examined. Under superfusion with HKRB without added Ca^2+^ and with 0.1 mM EGTA (Ca^2+^-free HKRB), administration of Ninjin'yoeito (10 μg/ml) for 10–12 min did not increase [Ca^2+^]_i_, while it subsequently increased [Ca^2+^]_i_ in HKRB with 2 mM Ca^2+^ (normal HKRB) in a single neuron that subsequently responded to ghrelin with [Ca^2+^]_i_ increase (Figure 2A). In nine ghrelin-responsive neurons, none responded to Ninjin'yoeito in Ca^2+^-free HKRB (Figure 2A, Right). This result indicated that the [Ca^2+^]_i_ response to Ninjin'yoeito in ghrelin-responsive neurons requires the presence of extracellular Ca^2+^.

We next examined whether particular type of VDCCs could be implicated in the [Ca^2+^]_i_ response to Ninjin'yoeito in ghrelin-responsive neurons. The effects of N-type Ca^2+^ channel blocker, ω-conotoxin, and L-type Ca^2+^ channel blocker, nitrendipine, were examined. In the presence of ω-conotoxin (100 nM) Ninjin'yoeito did not increase [Ca^2+^]_i_, while it subsequently increased [Ca^2+^]_i_ after washing out ω-conotoxin in the majority of single neurons (Figure 2B). In the presence of ω-conotoxin Ninjin'yoeito increased [Ca^2+^]_i_ in only 3 of 20 (15.0%) ghrelin-responsive neurons (Figure 2B, Right). By contrast, in the presence of nitrendipine (2 μM) Ninjin'yoeito increased [Ca^2+^]_i_ in the majority of single neurons (Figure 2C), and this response occurred in 21 of 23 (91.3%) of ghrelin-responsive neurons (Figure 2C, Right). These results indicated that Ninjin'yoeito increases [Ca^2+^]_i_ in the majority of ghrelin-responsive neurons via Ca^2+^ influx to which N-type Ca^2+^ channel has greater contribution than L-type Ca^2+^ channel.

### Ninjin-yoeito Increases [Ca^2+^]_i_ via Ca^2+^ Influx Preferentially Through L-Type VDCC in the Minority of Ghrelin-Unresponsive Neurons

**Figure 3:**
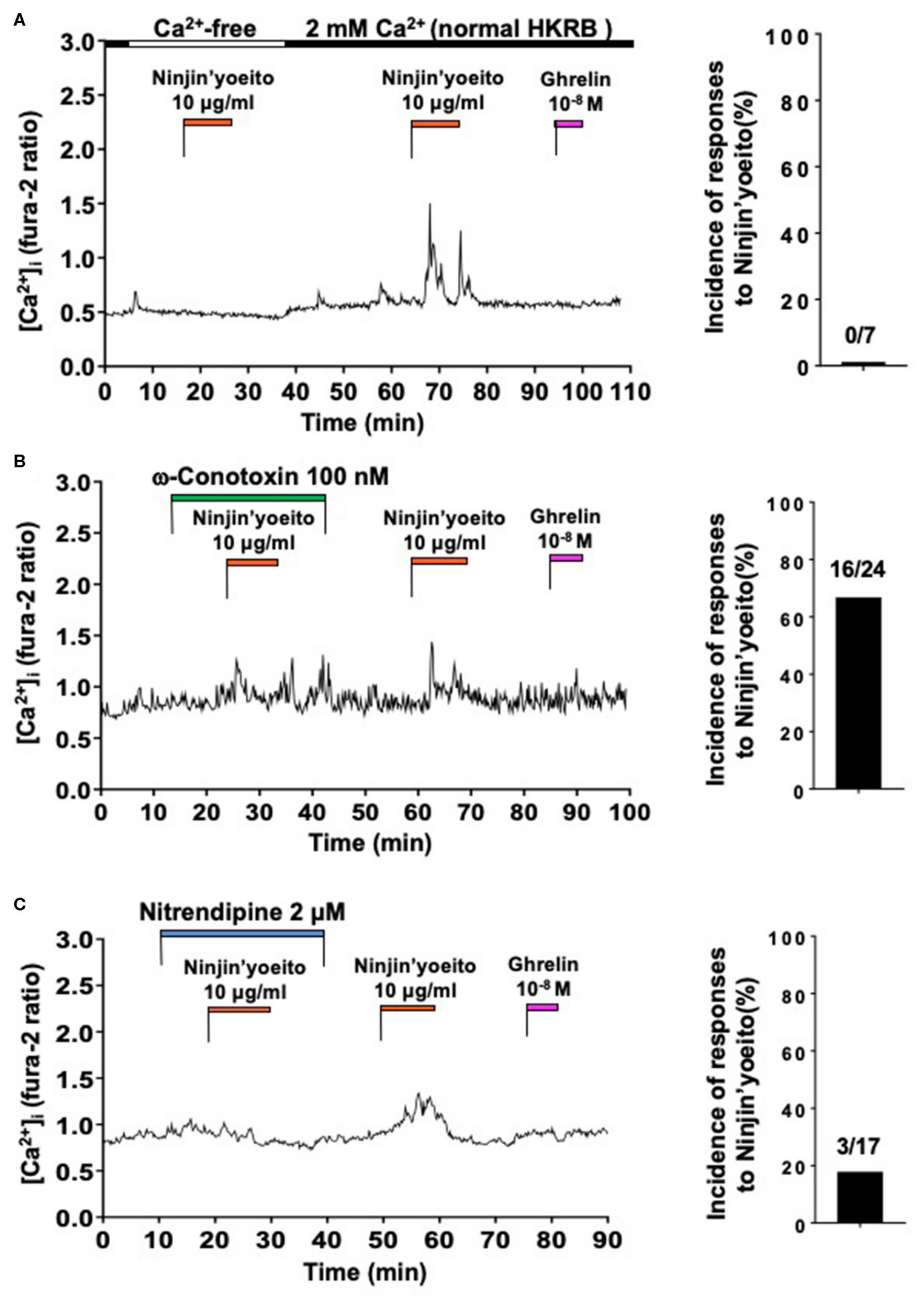
Ninjin'yoeito-induced [Ca^2+^]_i_ increases were inhibited in Ca^2+^ free condition and preferentially by L-type Ca^2+^ channel blocker in the majority of ghrelin-unresponsive ARC NPY neurons. ARC NPY neurons that responded to Ninjin'yoeito (10 μg/ml) but not to ghrelin (10^−8^ M) with increases in [Ca^2+^]_i_ were studied. Glucose concentration was 2.5 mM. **(A)** The [Ca^2+^]_i_ response to Ninjin'yoeito was inhibited under superfusion with Ca^2+^ free HKRB. Right: incidence (%) of responses to Ninjin'yoeito in Ca^2+^ free HKRB. Numbers above bar indicate number of neurons responding to Ninjin'yoeito over that examined. Data were from five preparations of single neurons from four mice. **(B)** An N-type Ca^2+^ channel blocker, ω-conotoxin, failed to inhibit the [Ca^2+^]_i_ response to Ninjin'yoeito. Right: incidence of responses to Ninjin'yoeito in the presence of ω-conotoxin. Numbers above bar indicate number of neurons responding over examined. Data were from six preparations of single neurons from three mice. **(C)** An L-type Ca^2+^ channel blocker, nitrendipine, inhibited the [Ca^2+^]_i_ response to Ninjin'yoeito. Right: incidence of responses to Ninjin'yoeito in the presence of nitrendipine. Numbers above bar indicate number of neurons responding over examined. Data were from nine preparations of single neurons from four mice.

The effect of Ninjin'yoeito (10 μg/ml) on [Ca^2+^]_i_ in ARC NPY neurons that did not respond to ghrelin were examined. Under superfusion with Ca^2+^ free KRB, Ninjin'yoeito did not increase [Ca^2+^]_i_ while it subsequently increased [Ca^2+^]_i_ in 2 mM Ca^2+^ KRB in a single neuron that subsequently failed to respond to ghrelin with [Ca^2+^]_i_ increase (Figure 3A). In nine ghrelin-unresponsive neurons, none responded to Ninjin'yoeito in Ca^2+^-free HKRB (Figure 3A, Right). This result indicated that the [Ca^2+^]_i_ response to Ninjin'yoeito in ghrelin-unresponsive neurons requires the presence of extracellular Ca^2+^.

We examined involvement of particular type of VDCC in the [Ca^2+^]_i_ response to Ninjin'yoeito in ghrelin-unresponsive neurons. Ninjin'yoeito induced increases in [Ca^2+^]_i_ both in the presence of and after washing out ω-conotoxin (100 nM) in the majority of single neurons (Figure 3B). The pattern and amplitude of [Ca^2+^]_i_ increases in response to Ninjin'yoeito in the presence and absence of ω-conotoxin were comparable (Figure 3B). In the presence of ω-conotoxin, Ninjin'yoeito increased [Ca^2+^]_i_ in 16 of 24 ghrelin-unresponsive neurons (66.7%) (Figure 3B, Right). By contrast, in the presence of nitrendipine (2 μM) Ninjin'yoeito failed to increase [Ca^2+^]_i_ in the majority of single neurons (Figure 3C), while it subsequently increased [Ca^2+^]_i_ after washing out this drug, showing a reversible inhibition. In the presence of nitrendipine Ninjin'yoeito increased [Ca^2+^]_i_ in only 3 of 17 ghrelin-unresponsive neurons (17.6%) (Figure 3C, Right). These results indicated that Ninjin'yoeito increases [Ca^2+^]_i_ in ghrelin-unresponsive neurons via Ca^2+^ influx to which L-type Ca^2+^ channel has greater contribution than N-type Ca^2+^ channel.

## Discussion

The present study employed [Ca^2+^]_i_ measurement in ARC single neurons combined with immunocytochemistry and found that among 43 ARC NPY neurons, 25 (58%) neurons responded to ghrelin and 18 (42%) neurons did not respond to ghrelin. This incidence of ghrelin-responsive neurons (58%) is not far from that in previous reports, considering different experimental conditions used among studies. The extracellular single unit recordings from *in vitro* slices indicated that ghrelin excited 73% of neurons in the ventromedial ARC, where NPY neurons are dominant, in adult rats (27). *Ex vivo* whole-cell patch-clamp recordings showed that ghrelin depolarized 40% of GFP-labeled arcuate NPY neurons in brain slices from 8–12 week-old male NPY-humanized Renilla reniformis green fluorescent protein transgenic mice (28). Ghrelin increased [Ca^2+^]_i_ in 59% of single ARC NPY neurons (9) and in 21–41% of single ARC neurons (12, 25) in 5–7 week-old male mice. The present study demonstrated that ghrelin-unresponsive neurons occur substantially among NPY neurons of ARC in 5–7 week-old male mice fed normal chow. Ninjin'yoeito-induced [Ca^2+^]_i_ increase was blunted in Ca^2+^-free condition in both ghrelin-responsive and ghrelin-unresponsive neurons. Ninjin'yoeito-induced [Ca^2+^]_i_ increases were inhibited by N-type Ca^2+^ channel blocker ω-conotoxin in the majority (17 of 20), while by L-type Ca^2+^ channel blocker nitrendipine in the minority (2 of 23), of ghrelin-responsive neurons. In contrast, Ninjin'yoeito-induced [Ca^2+^]_i_ increases were inhibited by nitrendipine in the majority (14 of 17), while by ω-conotoxin in the minority (8 of 24), of ghrelin-unresponsive neurons. Both N- and L-type Ca^2+^ channels are reportedly expressed and functioning in ARC (24, 25, 29). Our results demonstrate that Ninjin'yoeito increases [Ca^2+^]_i_ via Ca^2+^ influx, to which N-type Ca^2+^ channels have greater contribution in ghrelin-responsive neurons and L-type Ca^2+^ channels have greater contribution in ghrelin-unresponsive neurons. The [Ca^2+^]_i_ increase often results from membrane excitation and/or stimulated signal transduction and results in exocytosis, transport, gene expression, and/or protein regulation. Thus, the [Ca^2+^]_i_ increase, in general, reflects the neuronal activation. The present findings place N-type and L-type Ca^2+^ channels in ARC as potential molecular targets for Ninjin'yoeito to preferentially activate ghrelin-responsive and ghrelin-unresponsive NPY neurons, respectively, in ARC.

The mechanisms underlying the link of Ninjin'yoeito to distinct VDCCs in ghrelin-responsive and ghrelin-unresponsive neurons remain to be elucidated. It has been documented that ghrelin and/or GHSR influence the activities of VDCCs (30–36). Since ghrelin increases [Ca^2+^]_i_ via Ca^2+^ influx primarily through N-type Ca^2+^ channels in NPY neurons (25), it is speculated that Ninjin'yoeito could interact with the GHSR and/or downstream signaling linked to N-type Ca^2+^ channels in ghrelin-responsive NPY neurons. In consistent with this, it was previously reported that GHSR coexpression with dopamine type 2 receptor (D2R) reduces the inhibition of N-type VDCC currents by D2R activation (35). However, different results were also reported that N-type VDCC currents is inhibited by constitutive GHSR activity in mouse hippocampal cultures (34) and hypothalamic neurons (33). Though the cause for the apparent discrepancy among previous documents and our finding remains unknown, we investigated the effect of short-term (~5 min) administration of ghrelin in the current and previous studies (25), while some of previous documents observed the effect of constitutive GHSR activation (33, 34). Hence, ghrelin and GHSR signaling may have dual, acute stimulatory and chronic inhibitory, actions on N-type Ca^2+^ channel activity. Compared to ghrelin-responsive NPY neurons, properties of ghrelin-unresponsive ARC NPY neurons are less characterized. However, it was reported that a ligand for the taste receptor T1R2/T1R3 increased [Ca^2+^]_i_ in ARC neurons, the majority of which did not respond to ghrelin (26), and that this [Ca^2+^]_i_ increase was inhibited by nitrendipine, but not ω-conotoxin. Hence, T1R2/T1R3 is possibly linked to L-type Ca^2+^ channels in ghrelin-unresponsive ARC neurons and this pathway could be involved in the action of Ninjin'yoeito.

Though the Ninjin'yoeito's cellular signaling is less defined, it could interact with the excitatory signaling pathways in NPY neurons, which include the orexin—OX1R—phospholipase C pathway and low glucose—Na^+^,K^+^-ATPase suppression—depolarization pathway (37, 38). Notably, it was shown that AMPK activator AICAR increased [Ca^2+^]_i_ in two types of ARC NPY neurons, one with and the other without [Ca^2+^]_i_ responses to ghrelin (39), and that the AICAR-induced [Ca^2+^]_i_ increases were blunted in Ca^2+^-free conditions (40). Thus, Ninjin'yoeito and AICAR share the common properties: they stimulate Ca^2+^ influx in both ghrelin-responsive and unresponsive NPY neurons in ARC. In line with this, expression of carnitine palmitoyltransferase 1 (CPT1), a signaling molecule of AMPK, is regulated by metabolic conditions (41). Taken together, Ninjin'yoeito may elicit intracellular AMPK signaling pathway for activating ghrelin-responsive and ghrelin-unresponsive NPY neurons. However, further studies are definitely required to elucidate intracellular signaling mechanisms of Ninjin'yoeito.

The functional role of the Ninjin'yoeito-regulated VDCCs in ARC NPY neurons remains to be clarified. In ghrelin-responsive neurons, administration of Ninjin'yoeito interacts with N-type Ca^2+^ channels to enhance and/or cooperate with the action of ghrelin, possibly leading to efficacious activation of ghrelin-responsive NPY neurons and consequent stimulation of appetite. Our study places ARC N-type Ca^2+^ channel as a potential mediator and integrator of the actions of Ninjin'yoeito and ghrelin in ghrelin-responsive NPY neurons. On the other hand, the ghrelin-unresponsive ARC NPY neuron has been less defined for its physiological property and cellular lineage/differentiation. It has been documented that metabolic/feeding conditions induce dynamic remodeling of NPY/AgRP neurons and differentiation of feeding related neurons in ARC (42). Hence, the ghrelin-responsive neurons and ghrelin-unresponsive neurons could be converted to each other, depending on metabolic/feeding conditions and aging. Our finding that the type of VDCC correlates with ghrelin responsiveness in NPY neurons suggests that expression of specific VDCC type may be related to remodeling of NPY neurons. In this line, the ghrelin resistance, the phenomenon that ghrelin administration cannot stimulate feeding, occurs in association with aging and diet-induced obesity (21–23). This ghrelin resistance reportedly takes place in ARC NPY neurons (21), which could result in reductions in NPY neuronal activity and appetite. The transformation of ghrelin-responsive to ghrelin-unresponsive NPY neurons may underly ghrelin resistance. Of note, we found that Ninjin'yoeito preferentially target L-type VDCC to activate ghrelin-unresponsive NPY neurons in ARC. This action of Ninjin'yoeito could serve to compensate for the ghrelin resistance and restore appetite.

The present study demonstrated that Ninjin'yoeito activates the majority of ghrelin-responsive ARC NPY neurons preferentially via N-type VDCC while the majority of ghrelin-unresponsive NPY neurons preferentially via L-type VDCCs. We suggest ARC N-type VDCC as a target for activating ghrelin-responsive NPY neurons and promoting feeding while L-type VDCC as a target for activating ghrelin-unresponsive NPY neurons and possibly compensating for ghrelin-resistance to restore appetite.

## Data Availability Statement

The raw data supporting the conclusions of this article will be made available by the authors, without undue reservation, to any qualified researcher.

## Ethics Statement

Animal experiments were carried out after receiving approval from the Institutional Animal Experiment Committee and in accordance with the Institutional Regulation for Animal Experiments at Jichi Medical University and Kobe University.

## Author Contributions

TY designed the study. CG, KD, and LW conducted experiments. AI and YS participated in discussion. TY and CG wrote the manuscript. TY supervised the work. All authors contributed to the article and approved the submitted version.

## Conflict of Interest

TY received grant support from Kracie Pharma Ltd. provided Ninjin-yoeito but was not involved in the conducting of the current study at any stage. The remaining authors declare that the research was conducted in the absence of any commercial or financial relationships that could be construed as a potential conflict of interest.

## Abbreviations

VDCC: voltage-dependent Ca^2+^ channel
NPY: Neuropeptide Y
ARC: arcuate nucleus.
